# Chromosomal duplications and cointegrates generated by the bacteriophage lamdba Red system in *Escherichia coli *K-12

**DOI:** 10.1186/1471-2199-5-22

**Published:** 2004-12-13

**Authors:** Anthony R Poteete, Anita C Fenton, Ashwini Nadkarni

**Affiliations:** 1 Dept. of Molecular Genetics & Microbiology, University of Massachusetts Medical School, Worcester, MA 01655, USA

## Abstract

**Background:**

An *Escherichia coli *strain in which RecBCD has been genetically replaced by the bacteriophage λ Red system engages in efficient recombination between its chromosome and linear double-stranded DNA species sharing sequences with the chromosome. Previous studies of this experimental system have focused on a gene replacement-type event, in which a 3.5 kbp dsDNA consisting of the *cat *gene and flanking *lac *operon sequences recombines with the *E. coli *chromosome to generate a chloramphenicol-resistant Lac- recombinant. The dsDNA was delivered into the cell as part of the chromosome of a non-replicating λ vector, from which it was released by the action of a restriction endonuclease in the infected cell. This study characterizes the genetic requirements and outcomes of a variety of additional Red-promoted homologous recombination events producing Lac+ recombinants.

**Results:**

A number of observations concerning recombination events between the chromosome and linear DNAs were made: (1) Formation of Lac+ and Lac- recombinants depended upon the same recombination functions. (2) High multiplicity and high chromosome copy number favored Lac+ recombinant formation. (3) The Lac+ recombinants were unstable, segregating Lac- progeny. (4) A tetracycline-resistance marker in a site of the phage chromosome distant from *cat *was not frequently co-inherited with *cat*. (5) Recombination between phage sequences in the linear DNA and cryptic prophages in the chromosome was responsible for most of the observed Lac+ recombinants. In addition, observations were made concerning recombination events between the chromosome and circular DNAs: (6) Formation of recombinants depended upon both RecA and, to a lesser extent, Red. (7) The linked tetracycline-resistance marker was frequently co-inherited in this case.

**Conclusions:**

The Lac+ recombinants arise from events in which homologous recombination between the incoming linear DNA and both *lac *and cryptic prophage sequences in the chromosome generates a partial duplication of the bacterial chromosome. When the incoming DNA species is circular rather than linear, cointegrates are the most frequent type of recombinant.

## Background

The Red recombination system of bacteriophage λ promotes efficient double strand break repair/recombination. An *Escherichia coli *strain in which RecBCD has been genetically replaced by Red exhibits greatly elevated levels of recombination between its chromosome and short linear double-stranded DNA species sharing sequences with the chromosome [1].

**Figure 1:**
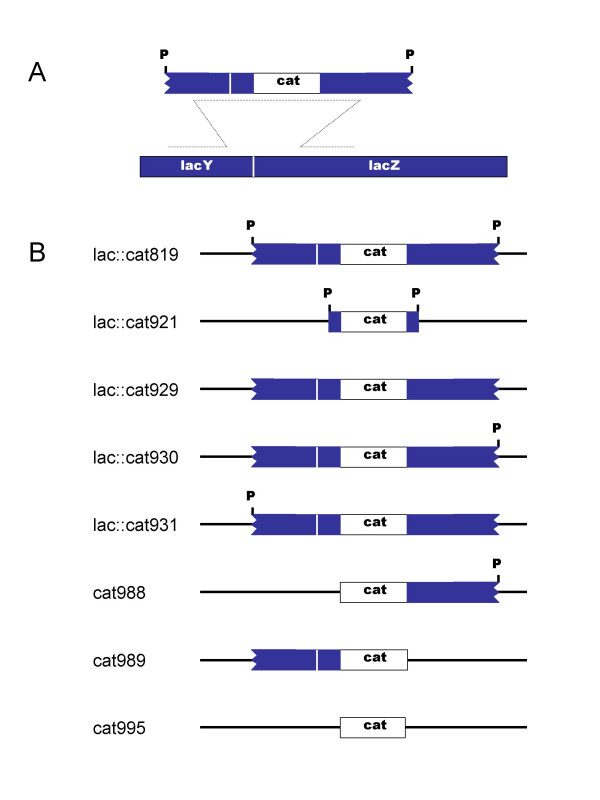
**λ *lac*::*cat *variants **A. Recombination between a 3.6 kbp linear dsDNA fragment and *lac *genes in the bacterial chromosome. A non-functional *lacZ*::*cat *allele is generated by the recombination event pictured. The linear DNA is released from the chromosome of a non-replicating λ phage, by the action of PaeR7 restriction endonuclease in the infected cell. The *lac *genes are shown as oriented in the conventional *E. coli *map. Their transcription is from right to left; replication forks travel through them from left to right. B. Structures of the *cat *substitutions in the λ phages used in this study. White bars represent *cat *and adjacent sequences from Tn9 (1 kbp). Colored bars represent *lac *sequences (1.3 kbp or 40 bp). PaeR7 sites are represented by P. All the substitutions replace the same λ sequences, bp 23,135–33,498, with the indicated sequences and an additional 1.9 kbp of sequence from phage P22 gene 9 (not shown in the diagrams). λ sequences replaced by the substitutions include the attachment site, *int*, *xis*, *exo*, *bet*, *gam, and cIII*. The substitutions result in net deletions of 5.0–7.6 kbp from the λ chromosome.

In previous studies, we have characterized a recombination event, pictured in Figure 1A, in which a 3.5 kbp dsDNA consisting of the *cat *gene and flanking *lac *operon sequences recombines with the *E. coli *chromosome to generate a chloramphenicol-resistant Lac- recombinant [2-4]. The dsDNA was delivered into the cell as part of the chromosome of a non-replicating λ vector, from which it was released by the action of the PaeR7 restriction endonuclease in the infected cell. Formation of Lac- recombinants was found to depend upon *red *and the bacterial recombination genes *recA*, *recF*, *recO*, *recR*, *recQ*, *ruvAB*, and *ruvC*. Large numbers of chloramphenicol-resistant Lac+ recombinants were generated in these crosses as well. In this study, we characterize the Lac+ recombinants and the processes which generate them. Most appear to arise from events in which homologous recombination between the incoming DNA and the chromosome generates a partial duplication of the bacterial chromosome.

## Results and discussion

### Genetic requirements for Lac+ recombinant formation

Data presented in Table 1 show that formation of Lac+ recombinants depends upon the same recombination functions as the Lac- recombinants: *recA*, *recF*, *recO*, *recR*, *recQ*, *ruvAB*, and *ruvC*. Deletion of *recG *increased the production of both Lac+ and Lac- recombinants. Lac+ recombinants accounted for 10–60% of the total chloramphenicol-resistant offspring of the crosses. These results strongly suggest that formation of the Lac+ recombinants takes place via homologous recombination, though they do not rule out the possibility that non-homologous end joining, or other varieties of "illegitimate" recombination, might be involved as well.

**Table 1:** Genetic requirements of Lac+ recombinant formation

Strain	Relevant genotype	Recombination	% Lac+
recG+	background		

507	wild type	1.00 ± 0.16	41
527	*recA*	0.01 ± 0.01	42
638	*recQ*	0.05 ± 0.02	37
540	*ruvAB*	0.34 ± 0.06	30
523	*ruvC*	0.22 ± 0.08	29

606	*sulA*	1.00 ± 0.31	40
608	*sulA lexA*	0.51 ± 0.01	44
615	*sulA recF*	0.12 ± 0.09	51
614	*sulA recO*	0.06 ± 0.00	42
625	*sulA recR*	0.03 ± 0.02	61

recGΔ	background		

554	wild type	1.00 ± 0.13	28
532	*recA*	0.01 ± 0.01	37
639	*recQ*	0.05 ± 0.03	46
559	*ruvAB*	0.34 ± 0.25	41
555	*ruvC*	0.08 ± 0.02	10

607	*sulA*	1.00 ± 0.14	37
609	*sulA lexA*	0.29 ± 0.12	22
628	*sulA recF*	0.03 ± 0.03	58
626	*sulA recO*	0.01 ± 0.01	21
627	*sulA recR*	0.02 ± 0.01	34

Bacterial strains were grown to log phase and infected at a multiplicity of 10 with λ *lac*::*cat819 nin5*. Infected cells were aerated for 1 hour at 37°C, then plated on LB agar and LB agar supplemented with chloramphenicol, isopropylthiogalactopyranoside, and X-Gal. Recombination frequencies represent the ratio of chloramphenicol-resistant recombinants to total cell titer, normalized to the ratio of the strain at the top of each group of strains. Means and standard errors for at least three measurements are shown. Actual frequencies were 507, 2.49%; 606, 2.50%, 554, 7.76%; 607, 7.81%. The frequencies of Lac- recombinants in these crosses were published previously (Poteete and Fenton, 2000).

The reason for considering mechanisms other than homologous recombination in the formation of the Lac+ recombinants is because of the expectation that some of the phage-borne *lac*::*cat *sequences would be attached to phage sequences. Heitman et al. [5] showed that double strand breaks generated by a restriction endonuclease *in vivo *are rapidly repaired by DNA ligase. The results of previous physical studies with other substituted λ phages cut *in vivo *by PaeR7 lead to the expectation that the λ *lac*::*cat819 *chromosome would be uncut, or only singly cut, much of the time in the infected cell [2,6].

Preliminary characterization of the Lac+ recombinants formed in cells which were wild-type for all the recombination functions revealed that most, possibly all, were unstable. When streaked on plates containing chloramphenicol and X-Gal, they segregated Lac- (colorless) progeny at variable frequencies. Southern gel analysis of chromosomal DNA from unstable Lac+ recombinants revealed that some of them were multiploid for *lacZ *(not shown). When the *lac*::*cat *dsDNA was delivered into the cell by a λ vector bearing a tetracycline resistance determinant (Δ *nin*::*tet859 *– described below) neither the Lac+ nor Lac- recombinants acquired tetracycline resistance. These findings suggest that the process which formed the recombinants did not involve the entire phage chromosome.

A baseline level of Lac+ recombinants was to be expected in these crosses. The recombination event pictured in Figure 1A, occurring in a cell with a pre-existing duplication of the *lac *locus, will produce a Lac+, chloramphenicol-resistant recombinant. Data presented below, in which simpler *lac*::*cat *recombining substrates generate Lac+ recombinants at a frequency approximately 1000-fold lower than Lac-, suggest the frequency of spontaneous *lac *duplications in Red+ but otherwise wild type *E. coli *is approximately 10^-3^, consistent with estimates of spontaneous duplication frequency in *Salmonella *made by Roth et al. [7]. The Lac+ recombinants formed in the crosses summarized in Table 1 occurred at a much higher frequency – approaching 5% of the infected cells – suggesting that pre-existing chromosomal duplications were not involved in the generation of the majority of the Lac+ recombinants. Apparently, normal Red-mediated homologous recombination between the phage and bacterial chromosomes duplicated or amplified sequences in the bacterial chromosome.

### Multiplicity effects

The crosses described above were done by infecting log phase host cells grown in rich medium. Such cells contain multiple copies of their chromosome. Complications might arise if the *lac*::*cat *segment were to recombine with more than one chromosome at a time. Further complicating interpretation of the experiment, the cells were infected at a multiplicity of 10 phages per cell.

To reduce the complexity of the system, we switched to a cross procedure involving low multiplicity infection (0.1 phage per cell) of stationary phase cells. Presumably, under these conditions, most of the events producing chloramphenicol-resistant recombinants take place between single copies of the *lac*::*cat *segment and single copies of the bacterial chromosome. As shown in Table 2, the low copy infections produced substantial numbers of Lac+ recombinants, though fewer than the high copy infections (4–7% of the chloramphenicol-resistant progeny versus 28–40%). The Lac+ colonies produced in this way were still unstable, segregating Lac- progeny (colorless colonies) when restreaked, or suspended and plated, on medium containing chloramphenicol and X-Gal (not shown).

**Table 2:** Recombinant formation by λ *lac::cat *variants

phage		description	host	genotype^a^	% recombinant	% Lac+
181	λ *lac*::*cat819*	cut both sides	507	wild	0.54 ± 0.06	7
197	λ *lac*::*cat930*	cut right	507	wild	0.92 ± 0.04	10
198	λ *lac*::*cat931*	cut left	507	wild	2.8 ± 0.07	0.2
196	λ *lac*::*cat929*	cut neither side	507	wild	0.055 ± 0.001	48
196	λ *lac*::*cat929*	cut neither side	839	*hsdR*	0.039 ± 0.002	29
181	λ *lac*::*cat819*	cut both sides	554	*recG*	0.93 ± 0.08	7
197	λ *lac*::*cat930*	cut right	554	*recG*	3.7 ± 0.04	13
198	λ *lac*::*cat931*	cut left	554	*recG*	4.6 ± 0.2	0.2
196	λ *lac*::*cat929*	cut neither side	554	*recG*	0.10 ± 0.03	47
186	λ *lac*::*cat819 nintet*		507	wild	0.28 ± 0.04	4
186	λ *lac*::*cat819 nintet*		849	*pae*	0.024 ± 0.003	45
186	λ *lac*::*cat819 nintet*		842	*pae hsdR*	0.027 ± 0.005	49
186	λ *lac*::*cat819 nintet*		850	*pae recA*	0.001 ± 0.002	95
186	λ *lac*::*cat819 nintet*		856	*pae red*	0.008 ± 0.008	52
208	λ *cat988 nintet*	right flank only	849	*pae*	0.008 ± 0.003	58
209	λ *cat989 nintet*	left flank only	849	*pae*	0.003 ± 0.0002	100
211	λ *cat995 nintet*	no flank	849	*pae*	0.004 ± 0.0002	100
195	λ *lac*::*cat921*	short flank	507	wild	0.002 ± 0.001	30
195	λ *lac*::*cat921*	short flank	554	*recG*	0.002 ± 0.0002	50

Bacterial strains were grown to stationary phase without active aeration, and infected at a multiplicity of 0.1 with the indicated phages. Recombination frequencies represent the percentages of infected cells which became chloramphenicol-resistant recombinants; % Lac+ refers to the percentage of chloramphenicol-resistant recombinants which were Lac+. Means and standard errors for at least three measurements are shown. a. All strains have the Δ*(recC*-*ptr*-*recB*-*recD)*::*P*_*tac*_-*gam*-*bet*-*exo*-*pae*-*cI822 *substitution, except as noted.

### Recombinant formation by single-cut variants of λ *lac*::*cat*

To examine the dependence of Lac- and Lac+ recombinant formation on the structure of the DNA substrate, we constructed variants of λ *lac*::*cat*, which are diagrammed in Figure 1. λ *lac*::*cat819*, the progenitor of this series, has two PaeR7 sites. Upon infecting a host cell bearing the Δ *recBCD*::*Ptac*-*gam*-*bet*-*exo*-*pae*-*cI *substitution, it injects its chromosome, which circularizes. Expression of its lytic genes, including those necessary for phage DNA replication, is blocked by the action of cI repressor. Cutting of its chromosome by the PaeR7 restriction endonuclease in the infected cell releases a 3.5 kbp linear dsDNA, consisting of the *cat *gene and 1.3 kbp flanks of *lac *sequences on the right and left. (In this and subsequent descriptions, "right" refers to the upstream, or 5' end of the *lac *operon). The ends of the flanking *lac *sequences match precisely their counterparts in the chromosome.

λ *lac*::*cat930 *and λ *lac*::*cat931 *have only single PaeR7 sites, on the right and left, respectively. Cutting by PaeR7 in the infected cell should produce large linear dsDNAs related to the *lac*::*cat819 *fragment, but with long tails of non-homologous DNA extending from the left and right sides, respectively. Interestingly, both of these single-cut phages produce recombinants more efficiently than the double-cut λ *lac*::*cat819*, in both recG+ and Δ recG backgrounds (Table 2). The right-cut λ *lac*::*cat930 *produces relatively more Lac+ recombinants, whereas the left-cut λ *lac*::*cat931 *produces fewer. The frequency of Lac+ λ *lac*::*cat931 *recombinants, 0.2%, is roughly consistent with the expected frequency of pre-existing *lac *duplications in the population of infected cells, suggesting that λ *lac*::*cat931 *does not generate duplications, but λ *lac*::*cat930 *does. We cannot rule out the possibility that some of the 0.2% Lac+ recombinants made by λ *lac*::*cat931 *are cointegrates. Cointegrate formation is discussed further in the next section.

### Recombinant formation by circular λ *lac*::*cat*

λ *lac*::*cat929 *has no PaeR7 site. Its chromosome should stay circular in the infected Δ *recBCD*::*Ptac*-*gam*-*bet*-*exo*-*pae*-*cI *cell. Surprisingly, it produces chloramphenicol-resistant recombinants only 10-fold less efficiently than λ *lac*::*cat819*. Unlike the PaeR7-cuttable λ *lac*::*cat *variants, λ *lac*::*cat929 *also efficiently produces weakly chloramphenicol-resistant microcolonies (not counted in the data presented in Table 2). The provenance of the microcolonies is readily understandable. The uncuttable λ *lac*::*cat929 *chromosome cannot replicate; it also is not destroyed, and should produce chloramphenicol acetyl transferase in the cell in which it resides. The infected cell, and perhaps its descendants for one or two generations, might be able to divide in the presence of low-concentration chloramphenicol.

Approximately half of the strongly chloramphenicol-resistant recombinants generated by the uncuttable phage λ *lac*::*cat929 *are Lac- (Table 2). This observation suggests that they are "legitimate" recombinants, in which the single chromosomal copy of *lacZ *is replaced by *lacZ*::*cat*. These recombinants were unexpected. All Red-mediated recombination events are thought to involve at least one linear partner [6]. How, then, can circular λ *lac*::*cat929 *recombine with the circular *E. coli *chromosome? Three possible explanations were considered. (1) Some of the λ *lac*::*cat929 *chromosomes might be cut by the EcoK restriction endonuclease. The infected cells contained a functional EcoK restriction-modification system. λ *lac*::*cat929 *was grown in a similarly EcoK+ host, and therefore was presumably EcoK-modified. However, if the modification were not complete, there might be residual EcoK restriction activity, resulting in 10% of the phage chromosomes being cut. (The phage chromosome contains five EcoK sites. If each site were 98% methylated, then approximately one in ten phage chromosomes would have a single unmethylated site). (2) The production of the recombinants might not be Red-mediated. (3) The linear partner might be a broken bacterial chromosome.

To test the first explanation, we replaced the gene encoding the EcoK restriction endonuclease, *hsdR*, with a tetracycline resistance determinant. This replacement had little effect on the formation of recombinants by λ *lac*::*cat929 *(Table 2).

To explore further the origins of the recombinants formed between circular phage chromosomes and the bacterial chromosome, we constructed a series of bacterial strains bearing variants of the Δ *recBCD*::*Ptac*-*gam*-*bet*-*exo*-*pae*-*cI *substitution lacking *pae*. In these hosts, no cutting takes place at PaeR7 sites, and so all of the *lac*::*cat*-substituted phages should remain circular. The results of crosses in these strains are shown in Table 2. As in the Pae+ crosses, elimination of *hsdR *in the Pae- background did not reduce the yield of recombinants. In the Pae- background, elimination of *recA *reduced the yield of recombinants twenty-fold. The great majority of the residual recombinants were Lac+, suggesting that their formation was by a process not necessarily involving homologous recombination. Elimination of *red *reduced the yield of recombinants three-fold, showing that most, but not all of the recombination events were Red-mediated. As in the Red+ cross, approximately half of the recombinants were Lac-, indicating that formation of both Lac- and Lac+ recombinants was Red-mediated in approximately the same proportions. Thus, the second hypothesis mentioned above, that the recombinants are formed by a Red-independent process, is not supported. The third hypothesis, that the linear partner might be a broken bacterial chromosome, is thus favored. It additionally seems reasonable, given that spontaneous double-strand breaks are frequent in *E. coli *[8].

We sought to detemine what proportion of the circular phage-by-chromosome recombinants were cointegrates, by testing for co-inheritance of *cat *and a tetracycline resistance-conferring element present at a remote location in the phage chromosome. Among the Lac+ recombinants produced in the Pae+ host TP507 (Table 2), only 2 out of 39 tested were found to be tetracycline-resistant; none of the 40 Lac- recombinants we tested was tetracycline-resistant. This result was consistent with our earlier finding that the great majority of recombinants formed in the high mulitiplicity infections of log phase cells acquired only the homology-flanked *cat *segment of the infecting λ chromosome. In contrast, in the Pae- host, 27 of 55 Lac- recombinants, and 23 of 25 Lac+ recombinants, were additionally tetracycline-resistant. These observations indicate that the most frequent Red-generated product of recombination between the uncut phage chromosome and the bacterial chromosome is a cointegrate.

**Figure 2:**
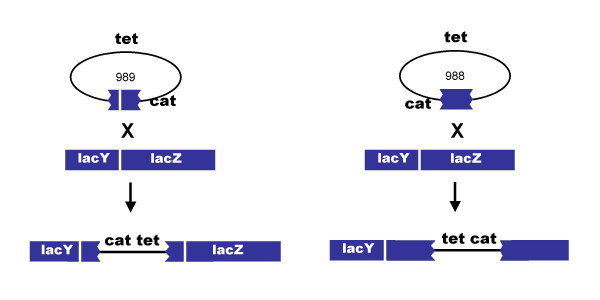
**Cointegrates formed by recombination between circular λ cat and the bacterial chromosome. **A. The cointegrate formed by recombination between the chromosome and λ *cat989*, which bears only the left-side *lac *flank, leaves the chromosomal *lacZ *gene intact. B. The cointegrate formed by recombination between the chromosome and λ *cat988*, which bears only the right-side *lac *flank, disrupts the chromosomal *lacZ *gene.

Cointegrates could in principle be formed by four different single reciprocal recombination events between the circular λ *lac*::*cat *and bacterial chromosomes. These events could involve the right side *lac *sequences or the left side *lac *sequences, as diagrammed in Figure 2. A third event, not shown, involves recombination between the *cI *genes borne by both the phage and bacterial chromosomes (the latter at the substituted *recBCD *locus). A fourth event, also not shown, involves recombination between λ genes and homologues in cryptic prophages in the *E. coli *chromosome (discussed below). As suggested in the figure, cointegrates formed by recombination in the right-side *lac *flank are phenotypically Lac-, while recombination in the left-side *lac *flank (or other loci) forms Lac+ recombinants. To test this idea, we constructed variants of λ *lac*::*cat *missing either or both *lac *flanks; they are diagrammed in Figure 1. As shown in Table 2, in the non-cutting Pae- host, only the right flank-containing phage chromosome forms Lac- recombinants. The left-flank and no-flank phages form only Lac+ recombinants.

### Short-flank recombination

The ability of the λ Red system to promote recombination events involving short sequence homologies makes it particularly useful for genetic engineering. Yu et al. [9] reported that a linear *cat *cassette with 1000 bp homologous flanks was only 10-fold more efficient than one with 40 bp flanks. Their experiments were done with cells which had been subjected to heat shock and electroporation. To test the efficiency of short-flank recombination under less extreme conditions, we constructed a short-flank λ *lac*::*cat *variant.

The fragment released by PaeR7 from the chromosome of λ *lac*::*cat921 *consists of the *cat *gene flanked by 40 bp sequences corresponding exactly to the terminal 40 bp at each end of the 1.3 kbp flanks of λ *lac*::*cat819*. As shown in Table 2, λ *lac*::*cat921 *exhibits a several hundred-fold lower efficiency of recombinant formation than its long-flank counterpart, in both recG+ and Δ recG backgrounds. λ *lac*::*cat921 *was similarly inefficient in log phase cells (data not shown). These observations suggest that short-flank recombination may not be a significant activity of the Red system in nature. However, they do not speak to the question of whether long flanks work better because they provide a larger homology target for synapsis, or because they provide non sequence-specific protection, perhaps delaying exonucleolytic degradation of the recombining sequences long enough to permit recombination to take place.

### Recombination with inverted partners

**Figure 3:**
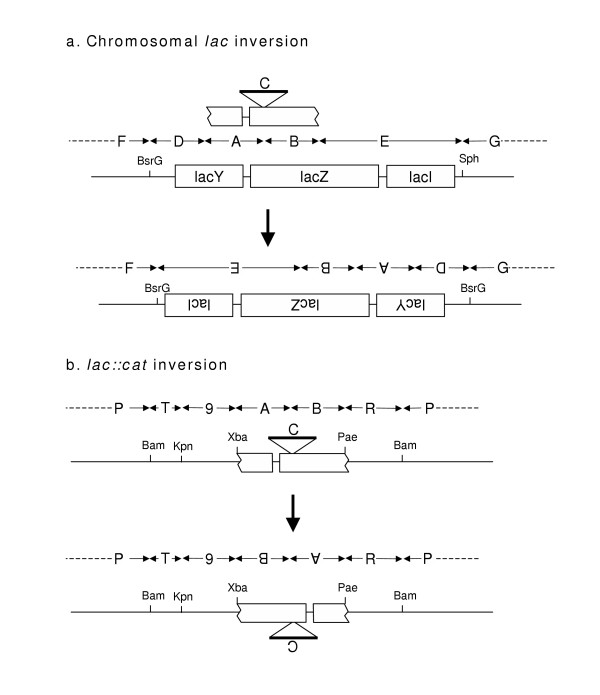
**Construction of inversions. **A. A plasmid containing the *lac *operon and some flanking sequences was constructed. The SphI site was converted to a BsrG1 site, the large BsrG1 *lac *segment was inverted, and the inverted operon was crossed into the bacterial chromosome. Sequence segments between certain boundaries – the inversion endpoints, the ends of the *lac *flanks, and the 12 bp of *lacZ *replaced by the *cat *gene – are given letter designations to simplify representations of parent and recombinant chromosomes in crosses described below. B. The structure of λ *lac*::*cat930 *is represented as a linear map. T designates the left side flanking λ sequences shared by plasmids and phages, consisting of λ bp 22346–23134. 9 designates phage P22 bp 17725–15955. R is the right side λ flank, bp 33502–34504. P is the rest of the phage λ chromosome. The *lac*::*cat *segment was inverted relative to the flanking sequences in plasmids pTP930 and 931 (generating pTP1032 and 1033), and the inverted elements were crossed into λ. The structure of λ *lac*::*cat1033 *is illustrated.

The observation that Lac+ recombinants are formed efficiently by the right side-cut λ *lac*::*cat930*, but not by the left side-cut λ *lac*::*cat931 *(Table 2, discussed above) raised questions concerning the sequence determinants of this directionality. To explore these questions, we constructed locally inverted variants of the bacterial chromosome and the phages, as diagrammed in Figure 3.

The strategy for inverting the chromosomal *lac *operon consisted of five steps (detailed in the Methods section and Table 4): (1) deletion of the entire operon from the chromosome, replacing it with the *cat *gene; (2) cloning the *cat *gene from the deletion mutant, along with flanking chromosomal sequences; (3) using the cloned, plasmid-borne flanking sequences, and Red-mediated gap repair, to clone the *lac *operon in a plasmid; (4) inverting the *lac *operon, relative to its flanking sequences, in the plasmid; (5) replacing the Δ *lac*::*cat *chromosomal allele with the plasmid-borne *lac*-*inv *allele.

**Table 4:** Bacterial strains used in this study

Strain	Relevant Genotype	Source, reference, or construction
AB1157 Background^a^		
KM22	Δ(*recC-ptr-recB-recD*)::*P*_*lac*_*-gam-bet-exo-kan*	Murphy, 1998
KM32	Δ(*recC-ptr-recB-recD*)::*P*_*tac*_*-gam-bet-exo-cat*	Poteete et al., 1999
TP507	Δ(*recC-ptr-recB-recD*)::*P*_*tac*_*-gam-bet-exo-pae-cI822*	"
TP523	Δ(*recC-ptr-recB-recD*)::*P*_*tac*_*-gam-bet-exo-pae-cI822 ruvC53 eda*::Tn *10*	Poteete and Fenton, 2000
TP527	Δ(*recC-ptr-recB-recD*)::*P*_*tac*_*-gam-bet-exo-pae-cI822 *Δ(*srl-recA*)*306*::*Tn10*	"
TP532	Δ(*recC-ptr-recB-recD*)::*P*_*tac*_*-gam-bet-exo-pae-cI822 recG258*::*kan *Δ(*srl-recA*)*306*::Tn *10*	"
TP540	Δ(*recC-ptr-recB-recD*)::*P*_*tac*_*-gam-bet-exo-pae-cI822 *Δ *ruvAB6203*::*tet*	"
TP554	Δ(*recC-ptr-recB-recD*)::*P*_*tac*_*-gam-bet-exo-pae-cI822 *Δ *recG6202*	"
TP555	Δ(*recC-ptr-recB-recD*)::*P*_*tac*_*-gam-bet-exo-pae-cI822 *Δ *recG6202 ruvC53 eda*::*Tn10*	"
TP559	Δ(*recC-ptr-recB-recD*)::*P*_*tac*_*-gam-bet-exo-pae-cI822 *Δ *recG6202 *Δ *ruvAB6203*::*tet*	"
TP606	Δ(*recC-ptr-recB-recD*)::*P*_*tac*_*-gam-bet-exo-pae-cI822 *Δ *sulA6209*::*tet*	"
TP607	Δ(*recC-ptr-recB-recD*)::*P*_*tac*_*-gam-bet-exo-pae-cI822 *Δ * recG6202 Δ sulA6209*::*tet*	"
TP608	Δ(*recC-ptr-recB-recD*)::*P*_*tac*_*-gam-bet-exo-pae-cI822 *Δ * sulA6209*::*tet lexA71*::*Tn5*	"
TP609	Δ(*recC-ptr-recB-recD*)::*P*_*tac*_*-gam-bet-exo-pae-cI822 *Δ *recG6202 Δ sulA6209*::*tet lexA71*::*Tn5*	"
TP614	Δ(*recC-ptr-recB-recD*)::*P*_*tac*_*-gam-bet-exo-pae-cI822 *Δ * sulA6209*::*tet recO1504*::*Tn5*	"
TP615	Δ(*recC-ptr-recB-recD)*::*P*_*tac*_*-gam-bet-exo-pae-cI822 *Δ * sulA6209*::*tet recF400*::*Tn5*	"
TP625	Δ *(recC-ptr-recB-recD*)::*P*_*tac*_*-gam-bet-exo-pae-cI822 *Δ * sulA6209*::*tet recR252*::*Tn10–9kan*	"
TP626	Δ(*recC-ptr-recB-recD*)::*P*_*tac*_*-gam-bet-exo-pae-cI822 *Δ *recG6202 Δ sulA6209*::*tet recO1504*::*Tn5*	"
TP627	Δ(*recC-ptr-recB-recD*)::*P*_*tac*_*-gam-bet-exo-pae-cI822 *Δ * recG6202 Δ sulA6209*::*tet recR252*::*Tn10–9kan*	"
TP628	Δ(*recC-ptr-recB-recD*)::*P*_*tac*_*-gam-bet-exo-pae-cI822 *Δ * recG6202 Δ sulA6209*::*tet recF400*::*Tn5*	"
TP638	Δ(*recC-ptr-recB-recD*)::*P*_*tac*_*-gam-bet-exo-pae-cI822 *Δ * recQ6216*::*tet*	"
TP639	Δ(*recC-ptr-recB-recD*)::*P*_*tac*_*-gam-bet-exo-pae-cI822 *Δ *recG6202 Δ recQ6216*::*tet*	"
TP839	Δ(*recC-ptr-recB-recD*)::*P*_*tac*_*-gam-bet-exo-pae-cI822 *Δ *hsdR*::*tet*	TP507 × PCR product^b^
TP842	Δ(*recC-ptr-recB-recD*)::*P*_*tac*_*-gam-bet-exo-cI978 Δ hsdR*::*tet*	TP849 × P1(TP839)
TP849	Δ(*recC-ptr-recB-recD*)::*P*_*tac*_*-gam-bet-exo-cI978*	KM22 × pTP978 linear^b^
TP850	Δ(*recC-ptr-recB-recD*)::*P*_*tac*_*-gam-bet-exo-cI978 *Δ *recA6207*::*tet*	TP849 × P1(TP796)
TP856	Δ(*recC-ptr-recB-recD*)::*P*_*tac*_*-gam-cI996*	KM32 × pTP996 linear^b^
MG1655 Background		
TP798	Δ(*recC-ptr-recB-recD*)::*P*_*tac*_*-gam-bet-exo-cat*	MG1655 × P1(KM32)
TP829	Δ(*recC-ptr-recB-recD*)::*P*_*tac*_*-gam-bet-exo-pae-cI822*	TP798 × pTP822 linear^b^
TP832	Δ(*recC-ptr-recB-recD*)::*P*_*tac*_*-gam-bet-exo-bla979*	TP798 × pTP979 linear^b^
TP872	Δ(*recC-ptr-recB-recD*)::*P*_*tac*_*-gam-bet-exo-bla979 * Δ * lac*::*cat*	TP832 × PCR product^b^
TP890	Δ(*recC-ptr-recB-recD*)::*P*_*tac*_*-gam-bet-exo-pae-cI822 *Δ * lac*::*cat*	TP829 × P1(TP872)
TP894	Δ(*recC-ptr-recB-recD*)::*P*_*tac*_*-gam-bet-exo-pae-cI822 lac-inv*	TP890 × pTP1034 linear^b^
TP896	Δ(*recC-ptr-recB-recD*)::*P*_*tac*_*-gam-bet-exo-bla979 lac-inv*	TP872 × pTP1034 linear^b^
MDS12 Background^c^		
TP750	Δ(*recC-ptr-recB-recD*)::*P*_*tac*_*-gam-bet-exo-pae-cI822*	Poteete, 2004
TP796	Δ(*recC-ptr-recB-recD*)::*P*_*tac*_*-gam-bet-exo-pae-cI822 *Δ * recA6207*::*tet*	"

a. These strains are presumed to bear all the other genetic markers of AB1157 (Bachmann BJ: Derivations and genotypes of some mutant derivatives of *Escherichia coli *K-12. In: *Escherichia coli and Salmonella: cellular and molecular biology*. Edited by Neidhardt FC, III RC, Ingraham JL, Lin ECC, Low KB, Magasanik B, Reznikoff WS, Riley M, Schaechter M, Umbarger HE. Washington, D.C.: ASM Press; 1996: 2460–2488).

b. Plasmids and PCR products used in the construction of some of the strains are described in the Methods section.

c. MDS12 is a derivative of wild type *E. coli *strain MG1655 bearing 12 large deletions (Kolisnychenko et al., 2002).

The single-cut *lac*::*cat930 *and *lac*::*cat931 *alleles were constructed by replacing the left-side and right-side PaeR7 sites, respectively, of pTP819, with XbaI sites. To produce inverted derivatives of these two alleles, the plasmids pTP930 and pTP931 were digested with XbaI and XhoI (a PaeR7 isoschizomer); *lac*::*cat *inserts and *bla*-*ori *backbone fragments from the two plasmids were exchanged. The inverted alleles were then crossed into phage λ, as described in the Methods section.

In constructing the chromosomal inversion, we switched genetic backgrounds, from the AB1157-derived strains with which most recombination studies have been done, to MG1655, the sequenced wild type *E. coli *K-12 [10]. In the MG1655 background, the same directionality of Lac+ recombinant formation was observed. The efficiencies of recombination of the MG1655 derivatives with both λ *lac*::*cat930 *and λ *lac*::*cat931*, and the efficiency of Lac+ recombinant formation by λ *lac*::*cat930*, were slightly elevated relative to those in the corresponding AB1157-derived strain.

**Figure 4:**
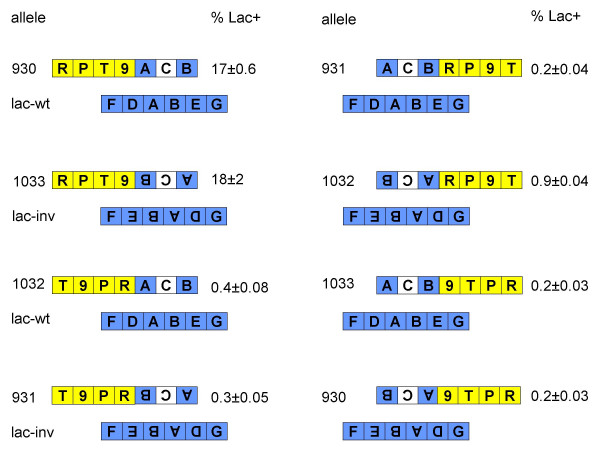
**Normal and inverted phage-by-chromosome crosses. **Phages bearing the indicated *lac*::*cat *alleles were crossed with wild type (TP829) and *lac*-*inv *(TP894) bacteria. Sequence segments are designated as shown in Figure 3. Bacterial sequences are colored blue, phage sequences yellow, and the *cat *gene white. The percentages of Lac+ bacteria among the chloramphenicol-resistant recombinant progeny are indicated, as the means and standard errors from three measurements. Crosses were done by low multiplicity infection of stationary phase cells. The total yields of chloramphenicol-resistant recombinants ranged from .02 to .04 per infected cell.

The eight combinations of normal and inverted phages and bacteria (left- and right-cut phages, and their inverted counterparts, in normal and *lac*-inverted *E. coli*) were tested for Lac+ recombinant formation. The results are shown in Figure 4. Only two of the eight combinations produced large numbers of Lac+ recombinants: *lac*::*cat930 *(right-cut, normal orientation) in *lac*-wild type, and *lac*::*cat1033 *(left-cut, inverted orientation) in *lac*-*inv*.

### Recombination with electroporated linear DNA species

**Figure 5:**
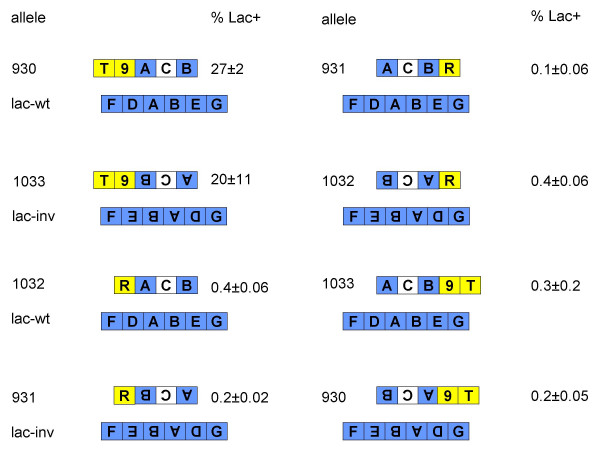
**Normal and inverted linear DNA-by-chromosome crosses. **Plasmids bearing the indicated *lac*::*cat *alleles (corresponding to the phages in Figure 4; the numbers of the plasmids bearing these alleles are given in the Methods section) were digested with BamHI and XhoI (PaeR7 isoschizomer), and electroporated into wild type and *lac*-*inv *bacteria (strains TP832 and TP896). Sequence segments are labeled as in Figure 3. The percentages of Lac+ bacteria among the chloramphenicol-resistant recombinant progeny are indicated, as the means and standard errors from two measurements. The total yields of chloramphenicol-resistant recombinants ranged from 20,000 to 200,000 per pmol.

The linear DNA produced by cutting of the λ *lac*::*cat *phages at a single site is large and complex relative to the *lac*::*cat *segment itself. To reduce the complexity, we generated linear DNA species which correspond to shortened versions of the single-cut phage chromosomes. The DNA species were generated by transferring, into a conditionally-replicating vector, parts of the plasmids previously used to introduce the *lac*::*cat *substitutions into λ. The vector can replicate only in a host which supplies the plasmid R6K Pir protein [11]. Details of the plasmid constructions are given in the Methods section. Plasmids bearing the cloned *lac*::*cat *and flanking sequences from λ were digested with restriction enzymes, and the DNA fragments were introduced into bacteria by electroporation. The results of some of these crosses are shown in Figure 5. The electroporated DNAs faithfully mimicked their single-cut phage counterparts: only the two crosses corresponding to the high Lac+ producer crosses of Figure 4 generated high proportions of Lac+ recombinants.

**Figure 6:**
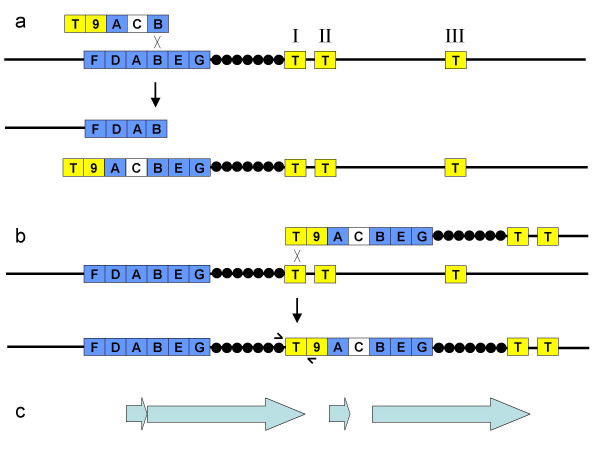
**Model for Lac+ recombinant formation in linear DNA-by-chromosome crosses. **(a) Recombination between the B sequence segments generates two broken chromosome arms. In the diagram, which is not to scale, I, II, and III designate the cryptic prophage *tfa *homologues *ybcX *in the DLP12 prophage remnant at 0.58 Mbp in the MG1655 chromosome, a closely linked unnamed pseudogene in the same element, and *tfaR *in the Rac prophage at 1.43 Mbp, respectively. The *lac *operon is at 0.36 Mbp. (b) The T segment-ended arm can recombine with any of the three T segments in an unbroken copy of the chromosome, generating a Lac+ chloramphenicol-resistant recombinant. Short arrows indicate the primers used to demonstrate the unique junction formed in the type I recombinant. (c) The recombinant contains two repeated sequence segments, a short one consisting only of the A segment (1.3 kbp), and a long one (0.2 or 1.1 Mbp) consisting of bacterial sequences from B through T.

The observation that only DNAs with sequences designated "T-9" on the left side generated many Lac+ recombinants (Figure 5) led us to try a linear species in which T was detached by digestion with a restriction enzyme (KpnI). Removal of the T segment greatly reduced the ability of the linear DNA to generate Lac+ recombinants (Table 3). A BLAST search of the *E. coli *genome for sequences related to T revealed four loci in MG1655 with close matches to T's leftmost 200 bp, which constitute the C-terminal third of the λ *tfa *gene. The four bacterial sequence segments are located in cryptic prophages: *ybcX *and an unnamed gene fragment in prophage DLP12, *tfaR *in prophage Rac, and *tfaQ *in prophage Qin. The locations and orientations of the first three of these are such as to permit a linear DNA species to recombine both with them and with the *lac *locus. Such an event is diagrammed in Figure 6, in which the three recombining bacterial loci are designated I, II, and III; closely related events have been extensively documented in other studies [7]. In the pictured cross, recombination between the linear DNA and the bacterial chromosome at *lac *and I generates a recombinant bearing intact *lac*, *lac*::*cat*, a duplication of all sequences between *lac *and I, a smaller duplication of the left *lac *flank, and an insertion of λ sequences unrelated to the cryptic prophages (see Figure 6c).

**Table 3:** Recombinant formation by variant linear DNA species

Plasmid	Restriction digest	Recombining linear species	% Lac+
1019	Bam + Xho	T9ACB	27 ± 2
1019	Bam + Xho + Kpn	9ACB	0.6 ± 0.2
1047	Bam + Xho	T9ACB(Δχ)	27 ± 2
1052	Bam + Xho	TACB	14 ± 0.1
1055	Sph + Xho	GCB	95 ± 3
1056	BsrG + Xho	ACF	99 ± 0.5
1057	Bam + Xho	TCB	96 ± 2
1018	Bam + Xho	ACB	0.2 ± 0.04

The indicated DNAs were introduced into TP832 by electroporation. DNA segments are labeled as in Figures 3–5. B(Δχ) is a variant of the 1.3 kbp right-side *lac *flank from which the 396 bp immediately adjacent to the *cat *segment (C) have been deleted, eliminating a Chi site. Figures indicate the means and standard errors from two measurements. Numbers in the first row are from Figure 5, top left, and are repeated here for comparison.

The hypothesis that the high-frequency Lac+ chloramphenicol-resistant recombinants are generated by homologous recombination with cryptic prophage sequences in the chromosome predicts that such recombinants would not be generated in an *E. coli *strain in which the cryptic prophages are deleted. To test this prediction, we electroporated linear *lac*::*cat930 *DNA into TP750, a Red+ derivative of MDS12, the reduced-genome *E. coli *strain constructed by Kolisnychenko et al. [12]. In this cross, Lac+ recombinants constituted only 0.06% (average of 6 measurements) of the total chloramphenicol-resistant progeny, a frequency no higher than expected for spontaneous pre-existing duplications in the bacterial chromosome.

**Figure 8:**
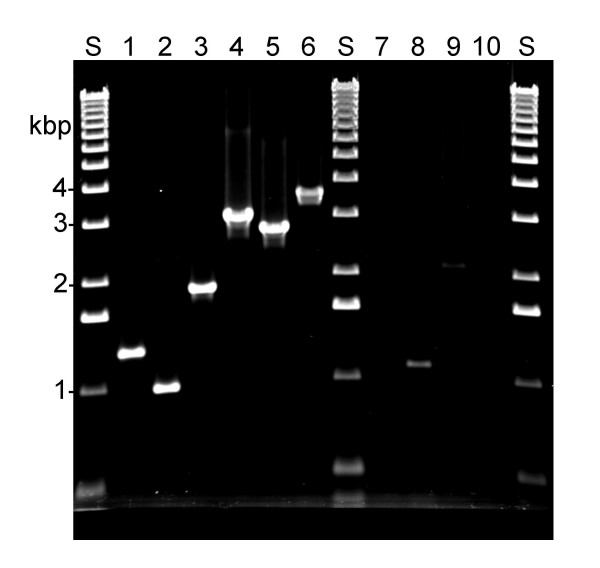
**PCR products indicating specific duplication junctions. **Colonies of Lac+ recombinants were tested by PCR as described in the Methods section. Lanes labeled S are standards (1 kb ladder, Invitrogen). Lanes 1, 2, and 3 are type I, II, and III recombinants, respectively, formed by electroporation of cells with the *lac*::*cat930 *DNA species diagrammed in Figure 5. The sizes of the expected products are: type I-1272 bp, II-1026 bp, III-1872 bp. Lane 4 is a recombinant formed by electroporation of cells with the GCB DNA species diagrammed in Figure 7. The size of the expected product is 3032 bp. Lane 5 is a recombinant formed by electroporation of cells with the ACF DNA species diagrammed in Figure 7. The size of the expected product is 2765 bp. Lane 6 is a recombinant formed by infection of cells with λ *lac*::*cat819*. The expected size of the product is 3653 bp. Lanes 7–10 are wild type cells used as template in control PCRs with the primers used in lanes 1–3, 4, 5, and 6, respectively.

The hypothetical Lac+ chloramphenicol-resistant recombinant pictured in Figure 6 has some predicted properties which were confirmed experimentally. First, it is predicted to segregate Lac- recombinants at low frequency, and chloramphenicol-sensitive recombinants at high frequency, the results of recombination between the short and long duplicated segments, respectively. Overnight cultures of three Lac+ recombinants grown in the absence of selection were found to include Lac- chloramphenicol-resistant clones at an average frequency of 0.03%, and Lac+ chloramphenicol-sensitives at 30%. Second, it should be possible to demonstrate specific duplication junctions in each of the three types of recombinants by the use of PCR (see Figure 6c). Primers were designed to this end, and employed in colony PCR with a collection of 12 Lac+ recombinants. Each of the 12 was found to have one of the three predicted junctions: seven were type I, two were type II, and three were type III. Examples are shown in Figure 8.

We constructed several other linear DNA substrates to test the generality of the model in Figure 6. The abilities of these substrates to generate Lac+ recombinants are shown in Table 3. The first of these was a derivative of *lac*::*cat930 *lacking its χ site. The χ site is located in the right *lac *flank, which is labeled "B" in Figures 3–6, close to the *cat *gene. The orientation of this χ site is such that it would be expected to interact productively with RecBCD enzyme approaching from the PaeR7- or Xho-generated right end of *lac*::*cat930*. While no activity of χ in these crosses was expected, as the bacteria lack RecBCD, the directionality of Lac+ recombinant formation was reminiscent of the directionality of χ-RecBCD interaction (see [13] for a review). The Δχ substrate was just as active as the χ + version, showing that χ does not contribute significantly to Lac+ recombinant formation. Similarly, a *lac*::*cat930 *derivative (pTP1052) lacking its sequences derived from phage P22 was equally proficient at generating Lac+ recombinants. A derivative missing the left *lac *flank made Lac+ recombinants almost exclusively; the small number of Lac- recombinants in this case probably represent cointegrates made by uncut plasmid DNA in the fragment preparation.

**Figure 7:**
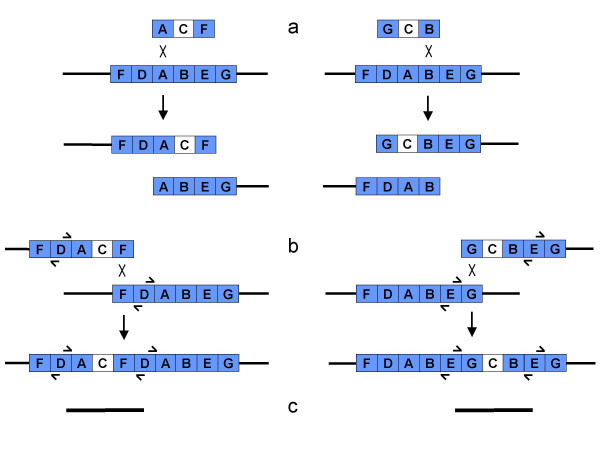
**Red-generated duplications to the left and right of lac. **Recombination between electroporated linear dsDNA species ACF (left) and GCB (right) generate duplications of chromosomal sequences to the left and right of *lac*, respectively, through a sequence of recombination events as pictured in Figure 6. (a) In the diagram, the first events are crossovers between the A or B *lac *segments, with crossovers between the associated F or G flanks pictured as occurring second (b). The same recombinants would also be formed if the orders were reversed, for example, if F recombined before A (not shown). Short arrows indicate the primers initiating DNA synthesis divergently from the D and E segments. (c) In the recombinants, the primers generate PCR products spanning the duplications, of 2765 and 3032 bp, respectively.

Plasmids pTP1055 and 1056 were constructed to test whether duplications of the type pictured in Figure 6 could be generated by Red-mediated recombination involving arbitrarily chosen sequences on either side of *lac *in the chromosome. As indicated in Table 3, linear DNAs from these plasmids also generated Lac+ recombinants almost exclusively. The structures of the plasmids, chromosome, and expected recombinants are diagrammed in Figure 7. That the expected duplications were in fact generated was demonstrated by the production of duplication-spanning products in PCR using the primers Ddi and Edi, described in the Methods section and represented schematically in Figure 7. Examples are shown in Figure 8.

### Structures of the λ *lac*::*cat *Lac+ recombinants

The major class of Lac+ chloramphenicol-resistant recombinants formed in crosses involving infection by λ *lac*::*cat819 *(cut left and right) and λ *lac*::*cat930 *(cut right) behave like their counterparts generated by electroporation of linear dsDNAs into Red+ cells: they segregate Lac- chloramphenicol-resistant clones at low frequency, and Lac+ chloramphenicol-sensitives at high frequency (data not shown). In addition, their formation also depends upon the presence of cryptic prophages in the chromosome: λ *lac*::*cat819 *was found to produce Lac+ chloramphenicol-resistant recombinants as only 0.13% (average of six measurements) of the total chloramphenicol-resistant progeny in crosses with TP750. These properties suggested both kinds of recombinant might have the same structures as well, but PCR tests of 50 of the phage-generated recombinants with the primers used to demonstrate type I, II, and III recombinants (Figure 8) were negative (data not shown).

Further computer analysis revealed a cryptic prophage sequence which could recombine with the phages, but not with the shorter, plasmid-derived linear DNAs, producing recombinants by the mechanism drawn in Figure 6: a 3.5 kbp patch of DLP12 closely matching sequences in the vicinity of the λ *cos *site. This site is located immediately to the left of the *tfaD *locus in the chromosome. Recombinants formed by crossing over at this site and at the right *lac *flank would be expected to contain duplications of bacterial sequences identical to those of type I recombinants (pictured in Figure 6), but to contain a larger part of the phage chromosome as well. PCR tests of six λ *lac*::*cat819 *and six λ *lac*::*cat930 *recombinants with primers designed to demonstrate *cos *recombinant junctions showed that all twelve had them. An example is shown in Figure 8.

The predominance of *cos *recombinants over *tfa *recombinants among the phage-generated Lac+ recombinants is to be expected. First, λ Exo, traveling in from the end of the long left-side non-homologous tail of λ *lac*::*cat930 *encounters *cos *before *tfa*; *cos *therefore presumably has a kinetic advantage. Second, the bacterial *cos *homology patch is significantly larger than the *tfa *homologies. A third possible advantage of *cos *is that, at least in some parts of the phage lytic cycle, it is the site of a double-strand break for the DNA encapsulation step of phage assembly. We expected that *cos *would be unbroken almost all the time in our crosses. The λ chromosome is linear at the time of injection, but is rapidly circularized by annealing and ligation [14]. The other parts of the phage lytic cycle are inhibited by the presence of cI repressor in the infected cells. Even so, the hypothesis that Red sometimes manages to gain access to *cos *ends in these crosses remains plausible; particularly so because Red is present in the cell at all times, whereas, in a normal infection, Red is not present until its genes are expressed from the phage chromosome. An unexpectedly significant presence of *cos *ends in the infected cells could also help explain the otherwise surprisingly high frequency of chloramphenicol-resistant recombinants seen in crosses involving phage chromosomes not cut by restriction endonucleases, described above.

One aspect of Lac+ recombinant formation by λ *lac*::*cat819 *is not explained by the model of Figure 6. The λ *lac*::*cat819 *contains two PaeR7 sites. Cutting by PaeR7 should release a linear dsDNA with *lac *flanks and no attached non-*lac *DNA. This DNA species did not generate Lac+ recombinants (above the background of pre-existing duplications) when electroporated directly into cells (Table 3). How does it apparently do so in the infected cells? As discussed above, it is expected that the λ *lac*::*cat819 *chromosome would be uncut, or only singly cut, much of the time in the infected cell. If it recombined at a time at which it was cut only on the right, the type of recombinant pictured in Figure 6 could be formed. In this recombinant, an uncut and unmodified PaeR7 site would sit in the chromosome. Presumably, the presence of this site would make the recombinant unstable, initially; but the PaeR7 site might eventually become modified, that is, escape restriction. The *pae*-expressing strains employed in these experiments restrict plaque formation by single PaeR7 site-bearing λ phages only approximately 5-fold (unpublished data).

## Conclusions

The bacteriophage λ Red recombination system is of general interest for two main reasons. First, it is an intensively characterized and relatively simple system, which serves as a model for studies of homologous recombination [15-17]. Second, it has emerged as a powerful tool for genetic engineering in gram-negative enteric bacteria [1,9,18-24]. It was therefore of interest to elucidate the structures of the previously observed but unexplained Lac+ recombinants formed by recombination between *lac*::*cat*-bearing λ phages and the bacterial chromosome, as well as the mechanism of their formation. Our studies uncovered a diversity of recombinant structures, including complex duplications and cointegrates. Recombination between λ and cryptic prophage sequences in the bacterial chromosome was found to be the most significant mechanism generating Lac+ recombinants. All of the Lac+ recombinants could be generated by homologous recombination events of types which have been previously described. In particular, there was no evidence for end-joining or other non-homologous recombination events. Perhaps the most surprising finding was the high frequency with which large duplications in the bacterial chromosome – up to 1 Mbp – could be generated by the Red system.

## Methods

### Bacteria

*E. coli *strain DH5α (λ pir) [11] was used for propagation of plasmids bearing the R6K origin. Other strains used in this study are described in Table 4. The Δ *hsdR*::*tet *allele in TP842 was constructed by using a Tn *10*-containing *E. coli *strain as template in PCR with primers hsdRut (5'-TTGGACAGGCCCGCACAGCAATGGATTAATAACAATGATGCTCGACATCTTGGTTACCGT-3') and hsdRdt (5'-GCTGAATTTGCCCAGCAGGGTATCGAGATTATCGTCAAAGCGCGGAATAACATCATTTGG-3'). The Δ *lac*::*cat *allele in TP872 was constructed by using a Tn *9*-containing *E. coli *strain as template in PCR with primers cat15 (5'-TCTGGTGGCCGGAAGGCGAAGCGGCATGCATTTACGTTGAATGAGACGTTGATCGGCACG-3') and cat16 (5'-AGAGTACATCTCGCCGTTTTTTCTCAATTCATGGTGTACAATTCAGGCGTAGCACCAGGC-3').

### Plasmids

#### Δ *nin::tet*

An EcoR1 fragment of λ *cI857 Sam7 *containing the *nin *region genes was cloned into the EcoR1 site of pBR322. In the resulting plasmid, pnin, the *nin *genes are read clockwise in the conventional map of pBR322. pTP772 was constructed by deleting sequences between the two HindIII sites of pnin. pTP859 was constructed by cutting pTP772 with SacII and ClaI, blunting the ends with T4 DNA polymerase, ligating with NotI linkers (5'-AGCGGCCGCT-3'), cutting with NotI, and ligating with a NotI fragment of pTP857 [25] containing the *tetR *and *tetA *genes of transposon Tn10.

#### *lac*::*cat *PaeR7 site variants

pTP819, in which the *cat *gene is flanked on both sides by *lac *sequences, PaeR7 sites, and λ sequences (for crossing into the phage), has been described [2]. pTP828 and pTP829 were described previously (without names) as intermediate plasmids in the construction of pTP819 [2]; each bears the cat gene with a single *lac *flank and PaeR7 site. pTP922 was constructed by deleting the *lac *and *cat *sequences between the two PaeR7 sites in pTP819. (In this and other plasmid constructions, PaeR7 sites were cut with XhoI, an isoschizomer). pTP926, pTP927, and pTP928 were constructed by ligating the oligonucleotide 5'-TCGACAGTCTAGACTG-3' into the PaeR7 sites of pTP828, pTP829, and pTP922, respectively, eliminating the PaeR7 sites and replacing them with XbaI sites. pTP929 was constructed by ligating the XbaI-NcoI fragment of pTP926 containing the N-terminal coding sequences of *cat *and the NcoI-XbaI fragment of pTP927 containing the C-terminal coding sequence of *cat *into the XbaI site of pTP928. The orientation of the reconstructed *cat *gene is the same as in pTP819. pTP930 was constructed by ligating together the large ApaI-SacII fragment of pTP929 and the small ApaI-SacII fragment of pTP819. pTP931 was constructed by ligating together the small ApaI-SacII fragment of pTP929 and the large ApaI-SacII fragment of pTP819.

#### pTP921 (short-flank *lac*::*cat*)

pTP921 was constructed by ligating together two XhoI-digested DNAs: pTP922 and a PCR product made by amplifying the *cat *gene from a Tn9-containing *E. coli *strain with primers 5'-GACGCACTCGAGGCGTTAACCGTCACGAGCATCATCCTCTGCATGGTCAGGCCGGCCACTGGAGCACCTCAAAAACACCA-3' and 5'-GACGCACTCGAGGCACACAGCGCCCAGCCAACACAGCCAAACATCCGCGCGGGCCCGACCGGGTCGAATTTGCTTTCGAA-3'. The presence of the expected sequences from the synthetic oligonucleotides in pTP921 DNA was verified by automated sequencing (data not shown).

#### *lac *flank variants

pTP988 (left *lac *flank only) was constructed by replacing the XbaI-ApaI *lacZY *segment of pTP930 with a mixture of two oligonucleotides, 5'-CTAGTTGCAAGCTTGGGCC-3' and 5'-CAAGCTTGCAA-3'. pTP989 (right *lac *flank only) was constructed by replacing the NgoMI-XhoI *lacZ *segment of pTP930 with a mixture of two oligonucleotides, 5'-CCGGCAAGCTTGCTGGTGGGCAA-3' and 5'-TCGATTGCCACCAGCAAGCTTG-3'. pTP995 (no *lac *flank) was constructed by ligating together the small NcoI fragment of pTP988 and the large, ori-containing NcoI fragment of pTP989.

#### Inverted *lac*::*cat*

pTP1032 was constructed by ligating together the XbaI- and PaeR7-ended *cat*-containing fragment of pTP930 and the XbaI- and PaeR7-ended *ori*-containing fragment of pTP931. pTP1033 was constructed by ligating together the XbaI- and PaeR7-ended *cat*-containing fragment of pTP931 and the XbaI- and PaeR7-ended *ori*-containing fragment of pTP930.

#### Inverted *lac*

pTP1016 was constructed by ligating into the NotI site of pTP809 [25] the NotI-digested PCR product made by amplifying the *cat *gene and flanking sequences from strain TP872 (Δ *lac*::*cat*) with primers 5'-CATCATCACGCGGCCGCGACGTTTGCCGCTTCTGAA-3' and 5'-ATCATCCACGCGGCCGCTGCGTTTTGCACCAGTACG-3'. In pTP1016, the *cat *gene is flanked closely by unique SphI and BsrGI sites. pTP1027 was constructed by electroporating SphI- and BsrGI-digested pTP1016 into strain TP829 (*lac+*); a plasmid formed by gap repair was isolated. pTP1028 was constructed by ligating SphI-digested pTP1027 with the oligonucleotide 5'-GTTGTACAACCATG-3', converting the SphI site into a BsrGI site. pTP1034 was constructed by cutting pTP1028 with BsrGI, ligating the two fragments back together, and screening for plasmids in which the *lac *genes were inverted relative to their flanking sequences.

#### R6K oriγ *lac::cat *plasmids

pTP1029, a tetracycline resistance bearing vector capable of replicating only in cells expressing R6K *pir *function, was constructed by ligating together two AatII- and Bam-digested DNA species: a PCR product made by amplifying a Tn10-containing E. coli strain with primers 5'-TCAACGTAAATGCATGGACGTCCTCGACATCTTGGTTACCGT-3' and 5'-TGTACACCATGAATTGGATCCCGCGGAATAACATCATTTGG-3'; and the *ori*-containing fragment of plasmid pLD54 [26]. pTP1018, 1019, 1020, 1044, and 1045 were constructed by ligating the BamHI *lac*::*cat*-containing fragments of pTP819, 930, 931, 1032, and 1033, respectively, into the BamHI site of pTP1029.

#### Rearranged *lac*::*cat *variants (Table 3)

pTP1047 was constructed by ligating the complementary oligonucleotides 5'-TCGTCTAGAGT-3' and 5'-CCGGACTCTAGACGAAGCT-3' between the SacI and NgoMIV sites of pTP1019. pTP1051 was constructed by ligating the complementary oligonucleotides 5'-CAGCATGCAT-3' and 5'-CTAGATGCATGCTGGTAC-3' between the KpnI and XbaI sites of pTP930. pTP1052 was constructed by ligating the complementary oligonucleotides 5'-CAGCATGCAGGGCC-3' and 5'-CTGCATGCTGGTAC-3' between the KpnI and ApaI sites of pTP1019. pTP1053 was constructed by ligating the complementary oligonucleotides 5'-GGCATGCAGGTTCTTTGAGTCCTTTGGGCGGCCGCGGGCC-3' and 5'-CGCGGCCGCCCAAAGGACTCAAAGAACCTGCATGCCGC-3' between the SacII and ApaI sites of pTP1019. pTP1054 was constructed by ligating the complementary oligonucleotides 5'-CCGGCGCGGCCGCAGGTTCTTTGAGTCCTTTGGTGTACAGAGCT-3' and 5'-CTGTACACCAAAGGACTCAAAGAACCTGCGGCCGCG-3' between the NgoMIV and SacI sites of pTP1020. pTP1055 was constructed by ligating the small SphI-NotI fragment of pTP1016 between the SphI and NotI sites of pTP1053. pTP1056 was constructed by ligating the small NotI-BsrGI fragment of pTP1016 between the NotI and BsrGI sites of pTP1054. pTP1057 was constructed by ligating the BamHI *cat*-containing fragment of pTP1051 into the BamHI site of pTP1029.

#### Δ *recBCD*::*Ptac-gam-bet-exo-pae-cI *variants

pTP822, which bears a synthetic *Ptac-gam-bet-exo-pae-cI *operon flanked by sequences upstream from *recC *on one side and sequences internal to *recD *on the other, has been described [2]. pTP978 was constructed by ligating together two NcoI- and XbaI-digested DNA species: pTP822, and a PCR product made by amplifying the *cI *gene from *E. coli *strain TP507 with the same primers used in the construction of pTPP822. This construction has the effect of simply deleting the *pae *genes from pTP822. pTP996 was constructed by deleting the C-terminal coding sequences of *bet *and the N-terminal coding sequences of *exo *between the two HpaI sites of pTP978. pTP979 was constructed by ligating together two NcoI- and XbaI-digested DNAs: pTP822 and a PCR product made by amplifying the *bla *gene from pBR322 with primers 5'-CCACCAATCATCCATGGCGCGGAACCCCTATTTGTTT-3' and 5'-TTGTTGGACGATCTAGAGGTCTGACAGTTACCAATGC-3'. This construction has the effect of replacing *pae *and *cI *with *bla*.

### Phages

λ *lac*::*cat819 *and λ *lac*::*cat819 nin5 *have been described [2]. λΔ *nin*::*tet859 *was made by crossing λ wild type with plasmid pTP859, infecting a tetracycline-sensitive strain with the resulting lysate, and selecting a tetracycline-resistant lysogen. The Δ *nin*::*tet859 *substitution replaces λ bp 40388–43825 with the *tetR *and *tetA *genes of transposon Tn *10*.

The *lac*::*cat921*, *929*, *930*, *931*, *988*, *989*, *995, 1032*, and *1033 *substitutions were crossed into λ wild type and/or λ *Δ nin*::*tet859*. The parent phages were crossed with the *cat *substitution-bearing plasmids. Phages which had acquired the substitution were either selected by plating on a strain lysogenic for phage P2 (Spi- phenotype), or identified by their clear-plaque morphologies (all the *cat *substitutions replace the *cIII *gene). The structures of the substituted phages were all verified by their ability to generate specific products when used as templates in PCR (not shown).

### Crosses

Phages were crossed with plasmids by spotting enough phage to make a confluent zone of lysis on a lawn of a sensitive bacterial strain bearing the parent plasmid. After overnight incubation at 37°C, material from the zone was collected in TM (10 mM Tris-HCl pH 7.5, 10 mM MgSO_4_), and shaken with chloroform. Two methods for crosses monitoring recombination between phage-injected DNA and the bacterial chromosome, both previously described, were employed: high-multiplicity infection of log phase cultures [27], and low-multiplicity infection of cells grown to stationary phase by standing overnight incubation [4].

Crosses involving recombination between bacterial chromosomes and electroporated DNA fragments were carried out as described by Murphy and Campellone [23]. The DNA fragments were generated by digesting various plasmids with restriction endonucleases. Amounts of DNA used varied among experiments, corresponding to 250–500 ng of the 3595-bp *lac*::*cat *Xho fragment; within an experiment, equimolar amounts of different recombining linear species were used. DNA was quantitated by running samples in an agarose gel, staining with ethidium bromide, and measuring fluorescence of the bands by the use of a Kodak Gel-Logic 200 system with 1-D software.

### PCR

The structures of various Red-generated duplications in the *E. coli *were verified by PCR. Colonies were picked and added to 40 μl mixtures containing 1 unit Taq polymerase, 63 μM dNTPs, 1.88 mM MgCl_2_, 5% (v/v) dimethylsulfoxide, 20 mM Tris-HCl pH 8.4, 50 mM KCl, and primers at 0.7 μM. Samples were heated to 95C for 5 min, then put through 30 cycles of 94C for 1 min, 55C for 1 min, 72C for 2–4 min, depending upon the length of the expected products. Primers for demonstrating the type I, II, and III *tfa *junctions were combined in a single mixture of 4 oligonucleotides: Jsp1 (GTTGAATGGGCGGATGCTAA), Jsp2 (TCTTCCACCAGAAAGCTACC), JncA (TGCCGTGTGAACGGTTTAC), and Jnc2 (TTCTAGCCCCATCATCTGTG). Primers for spanning the ACF-generated duplication (see Figure 7) were Ddi1 (CTCTTTCCGTTACGGGACAC) and Ddi2 (TGGTGAACATGATGCCGACA). Primers for spanning the GCB-generated duplication were Edi1 (CGCCGAAATCCCGAATCTCT) and Edi2 (ACCGGCATACTCTGCGACAT). Primers for demonstrating the DLP12 *cos *junction were Csj1 (GATTGAGCGTGAAGTCTGTTTGTG) and Csj2 (CGAATAGTCGGCTCAACGTGGGTT).

## Abbreviations

PCR: polymerase chain reaction. X-Gal: 5-bromo-4-chloro-3-indolyl-β-D-galactopyranoside. IPTG: isopropyl β-D-thiogalactopyranoside. bp: base pairs.

## Authors' contributions

ACF carried out the experiments summarized in Table 1. AN and ARP carried out the experiments summarized in Table 2. ARP carried out the other experiments and wrote the paper. ARP, ACF, and AN constructed the plasmids, phages, and bacterial strains.
